# P85 Epidemiology, resistance patterns and antibiotic treatment for *Staphylococcus aureus* bloodstream infections: a 10 year analysis in St George’s NHS Trust

**DOI:** 10.1093/jacamr/dlaf230.092

**Published:** 2025-12-04

**Authors:** Rebeca Galán-Baquero, Tim Planche, Catrin E Moore

**Affiliations:** Infection and Immunity, St George’s School of Health and Medical Sciences, City St George’s, University of London, London, UK; Infection and Immunity, St George’s School of Health and Medical Sciences, City St George’s, University of London, London, UK; Infection Care Group, St. George’s University Hospitals Healthcare NHS Trust, London, UK; Infection and Immunity, St George’s School of Health and Medical Sciences, City St George’s, University of London, London, UK

## Abstract

**Background:**

*Staphylococcus aureus* causes a wide variety of infections, ranging from simple skin and soft tissue infections to life-threatening bloodstream infections. *S. aureus* was associated with over 1.1 million deaths globally in 2019.^1^ In the United Kingdom *S. aureus* is the second most frequent cause of bloodstream infections with around 5000 deaths per year. Moreover, MRSA is listed a high-priority pathogen by the WHO, and when isolated from blood cultures is in the mandatory national surveillance programme.

**Objectives:**

To investigate the longitudinal trends in *S. aureus* bloodstream infections, resistance patterns and antibiotic prescribing at St George’s NHS Trust over a 10 year period.

**Methods:**

We conducted a retrospective analysis of laboratory and medical records from St George’s NHS Trust. All positive *S. aureus* blood cultures collected between January 2014 and May 2024 were included together with the susceptibility results for all antibiotics tested. Available pharmacy prescribing records were also reviewed (April 2017–May 2024) to evaluate antibiotic use by class, age group and year.

**Results:**

We identified 917 patients (941 cases) with a mean annual incidence of 85.6 cases (95% CI: 80.1–91.0). The majority were due to MSSA (93.7%) with a significant upward trend over the ten years (*P*=0.002), MRSA remained stable (5.36 cases/year; 95% CI: 2.77–7.95). The prevalence of fluoroquinolone-resistant strains increased most markedly, particularly for levofloxacin and moxifloxacin (*P*=0.001 and *P*=0.01) which rose from 0 to 12.50% and 12.58% respectively, particularly in MSSA isolates. A total of 16 134 antibiotic prescriptions were recorded for 537 patients over the 7 year period. Antibiotic prescribing largely aligned with NHS treatment guidelines, with penicillins prescribed predominantly (48.8%), and the most common of this group was flucloxacillin (37.6%). Regression analysis showed increasing use of penicillins, cephalosporins, carbapenems, tetracyclines, linezolid and ciprofloxacin, and a decreasing use of macrolides and teicoplanin over the timespan.

**Conclusions:**

MSSA incidence accounted for most *S. aureus* bloodstream infections in St George’s NHS hospital over the 10 year period and increased significantly over time, MRSA incidence remained stable. Fluoroquinolone resistance rose markedly, particularly for levofloxacin and moxifloxacin among MSSA isolates. Antibiotic prescribing patterns were broadly consistent with NHS guidelines, with increasing use of penicillins, linezolid and other classes, and declining use of macrolides and teicoplanin over the 10 years.
